# Data on mammary gland microRNAs expression, their predicted gene targets and corresponding pathway analysis in female mice receiving flaxseed or its oil and secoisolariciresinol diglucoside components

**DOI:** 10.1016/j.dib.2022.108328

**Published:** 2022-05-29

**Authors:** Diana Wu, Amel Taibi, Zhen Lin, Lilian U. Thompson, Elena M. Comelli

**Affiliations:** a Department of Nutritional Sciences, University of Toronto, ON, Canada; b Joannah and Brian Lawson Centre for Child Nutrition, University of Toronto, ON, Canada

**Keywords:** Flaxseed, Flaxseed oil, secoisolariciresinol diglucoside, microRNA, mammary gland, female mice, NanoString, target predictions

## Abstract

Dietary flaxseed may act via microRNAs (miRNAs) to affect the health of the mammary gland. These data are in support of the article entitled “Effects of flaxseed and its components on mammary gland miRNome: identification of potential biomarkers to prevent breast cancer development” [1]. Here, we provide miRNA expression data obtained from NanoString nCounter® profiling of mammary gland RNA from C57BL/6 female mice who received a control diet or isocaloric diets containing 10% FS, 3.67% FSO, or 0.15% SDG for 21 days. The raw miRNA data were deposited at the NCBI Gene Expression Omnibus (GEO) database (https://www.ncbi.nlm.nih.gov/geo/query/acc.cgi?acc=GSE193847) under the accession number GSE193847. We also identified diet-associated miRNA-gene targets and corresponding enriched pathways. These data can be found at the HARVARD Dataverse (https://doi.org/10.7910/DVN/3ZNYES). These data will be valuable as a reference to understand the effects of FS versus its components and to study responses to these ingredients in hosts of different genetic backgrounds, sex and age. These data will contribute to future investigations regarding mechanisms underlying FS effects within the mammary gland.

## Specifications Table

**Table:** 

Subject	Genetics: Epigenetics
Specific subject area	Nutrition, microRNAs, mammary gland development
Type of data	Table Figure Reporter Code Count (RCC) files corresponding to the raw miRNA data collected using the Nanostring digital analyzer Txt files extracted from the RCC files using the Nanostring nSolver software.
How the data were acquired	Expression profiling of 578 miRNA using the nCounter® Mouse v1.5 miRNA Expression Assay Kit (NanoString Technologies, Seattle, WA, USA; miRBase built v15) and a NanoString digital analyzer version 4.0.0.3 at the Princess Margaret Genomics Centre (Toronto, ON, CA)
Data format	Raw Analyzed Filtered
Description of data collection	Female C57BL/6 mice at 4-5 weeks of age were randomized into 4 groups to receive a control basal diet (BD), or a basal diet modified to contain 10% FS, or 3.67% FSO, or 0.15% SDG for 21 days. The diets were isocaloric and the amounts of FSO and SDG were at the same levels present in the FS diet. Mice were sacrificed at the end of the intervention and the fourth left mammary gland was excised for total RNA extraction and miRNA profiling using the nCounter® Mouse v1.5 miRNA Expression Assay Kit (NanoString Technologies, Seattle, WA, USA) (miRBase built v15) following the manufacturer protocol. Raw data were processed using nSolver^TM^ Analysis Software v4.0 (NanoString Technologies). This included background-correction and normalization to the mean calculated across all the probes that had been detected above the background in 80% of the samples. Samples with more than 25% of probes below the background were excluded from the analysis. The final dataset was log2 transformed and used for statistical analysis.
Data source location	Institution: University of Toronto City/Town/Region: Toronto Country: Canada Latitude and longitude for collected samples/data: 43.6629° N, 79.3957° W
Data accessibility	The raw and processed miRNA data were deposited in a public repository under the accession number GSE193847 Repository name: National Center for Biotechnology Information (NCBI) Gene Expression Omnibus (GEO) database Data identification number: GSE193847 Direct URL to data: https://www.ncbi.nlm.nih.gov/geo/query/acc.cgi?acc=GSE193847 The secondary data including gene targets predictions and pathways analysis are with this article and deposited in the HARVARD Dataverse Repository name: HARVARD Dataverse Data identification number: doi:10.7910/DVN/3ZNYES Direct URL to data: https://doi.org/10.7910/DVN/3ZNYES
Related research article	Taibi A, Lin Z, Tsao R, Thompson LU, Comelli EM. Effects of Flaxseed and Its Components on Mammary Gland MiRNome: Identification of Potential Biomarkers to Prevent Breast Cancer Development. Nutrients. 2019 Nov 4;11(11):2656. doi:10.3390/nu11112656.

## Value of the Data

• These data provide the largest profile of miRNA expression in the mammary gland of pubertal female mice receiving or not flaxseed, flaxseed oil and SDG. This is important because miRNA may be an underlying mechanism for mammary gland development.
• Scientists studying the mammary gland may use these data as a basis to understand its response to flaxseed and its components in hosts of different genetic backgrounds, sex and age. Dose-responses can also be investigated.
• Researchers can use these data for secondary analyses or to design new experiments targeting selected miRNAs.
• This data can be used to create computational tools estimating relationships between miRNA changes and consumption of flaxseed or other lignan-containing foods in relation to health and disease.

## 1 Data Description

Data were obtained as described in the supported manuscript [1]. Briefly, raw MG miRNA reads were obtained using nCounter® Mouse v1.5 miRNA Expression Assay Kit (NanoString Technologies, Inc., Seattle, WA, USA) (miRBase built version 15) and were deposited at the NCBI Gene Expression Omnibus (GEO) database under the Data ID GSE193847. After filtering, background subtraction, and data normalization, 238 miRNAs were identified as expressed in the mammary gland of mice in the BD, FS, FSO, and SDG groups [1]. The miRNA data were log2-transformed for use in further analyses. Ten miRNAs were uniquely deregulated in one of the dietary groups compared to the basal diet group as reported in [1] and for clarity they are listed here for each dietary group vs control: FS, miR-297, miR-500, miR-1, and miR-210; FSO, miR-30, miR-324-5p, miR-382, and miR-423-3p; and SDG, miR-142-5p and miR-1966.

**Fig. 1:**
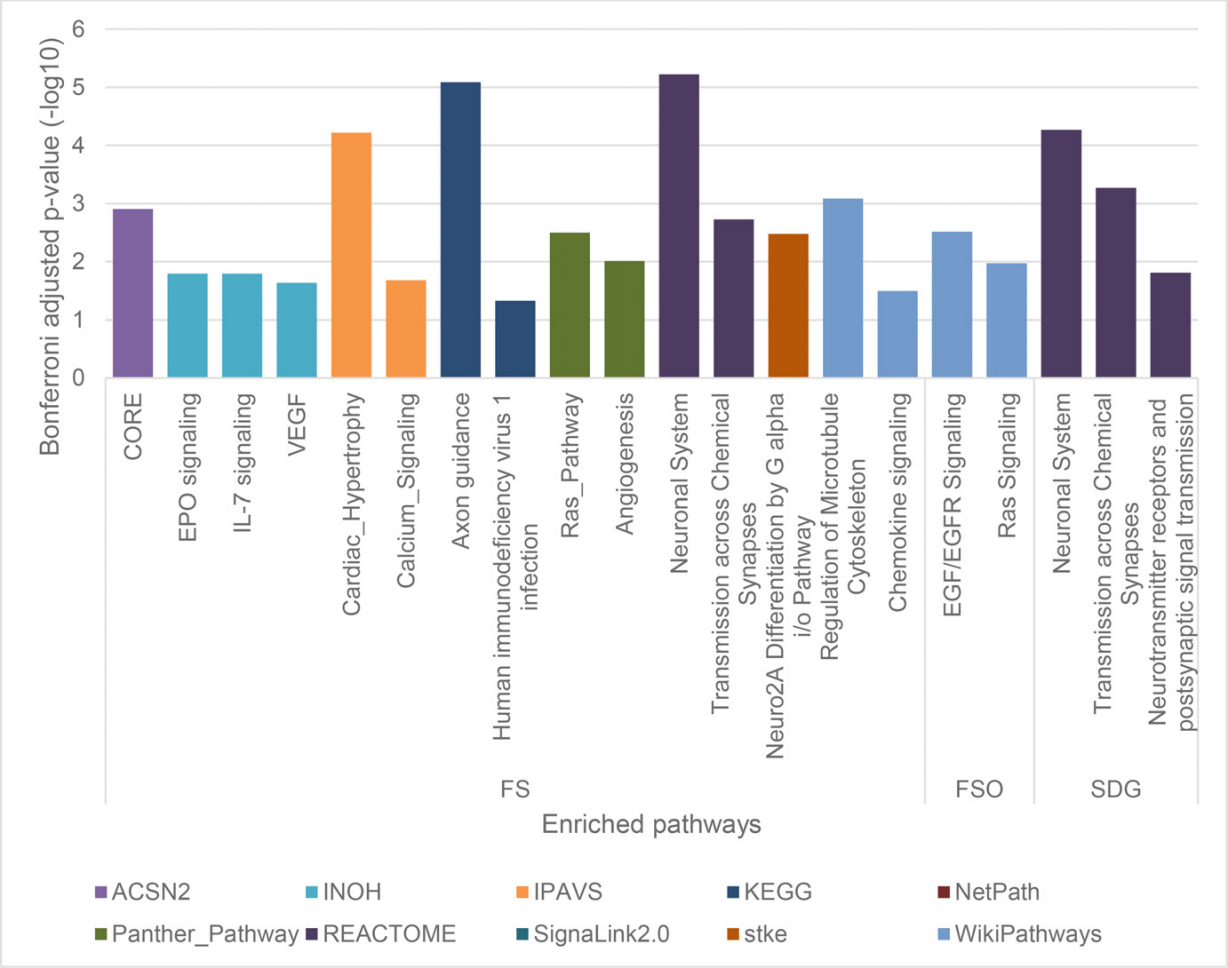
Enriched pathways identified *in silico* with pathDIP for FS diet, FSO diet, and SDG diet. Significance for each pathway is represented by adjusted p-value (Bonferroni-adjusted p-value), bar colours denote pathway sources.

**Fig. 2:**
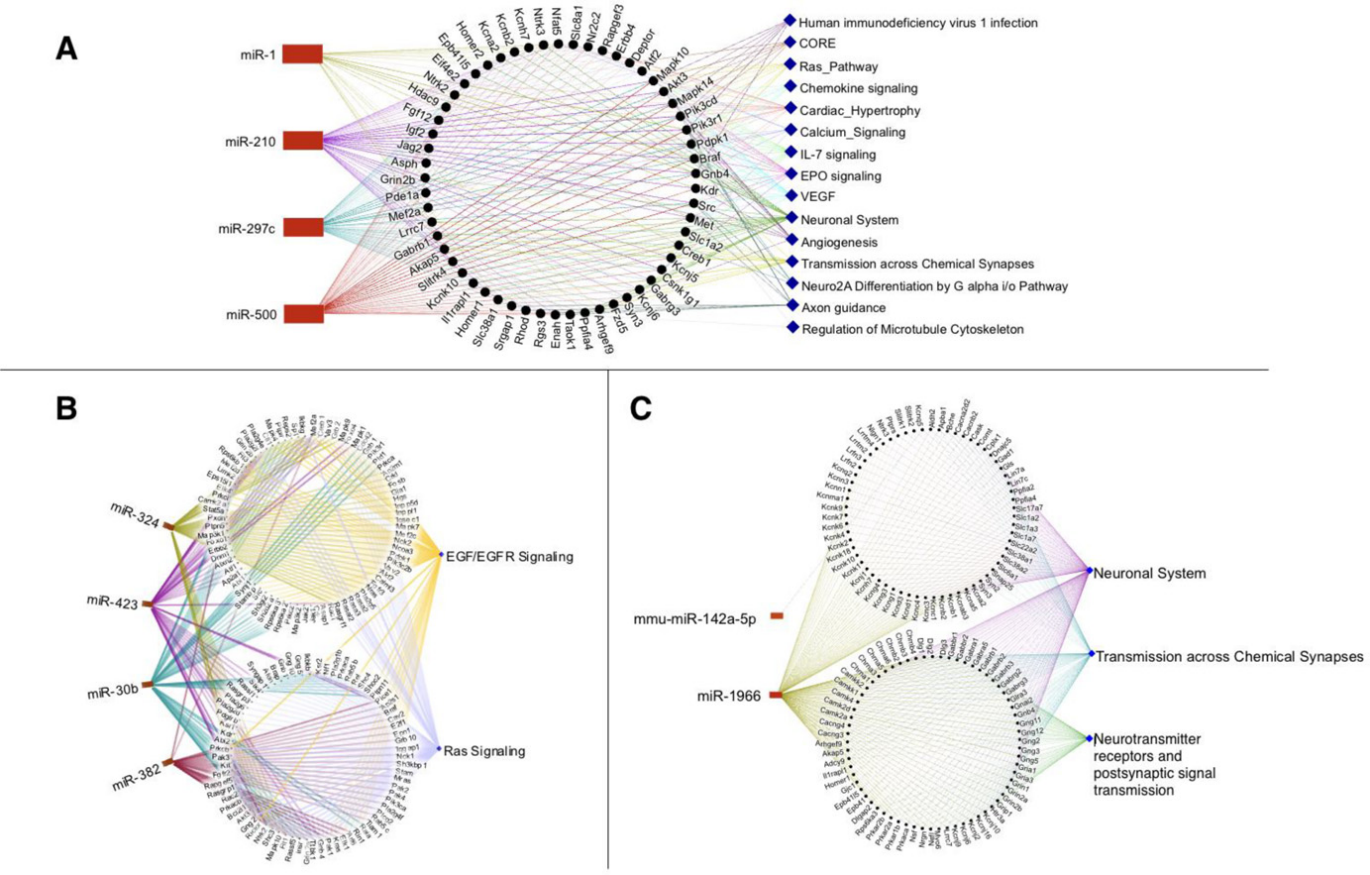
Predicted miRNA-gene target-pathway networks. Gene targets were identified with mIRWalk for miRNAs unique to A) FS diet, B) FSO diet, C) SDG diet. For interpretability, panel A contains only genes targeted by 3 or more miRNAs. Panels B and C contain all genes involved in enriched pathways. The full network figure for the FS diet can be found in Supplementary Fig. 1. MiRNAs are denoted in red and pathways are denoted in blue.

Supplementary Tables 1 and 2 list the gene targets of the diet-associated miRNAs as predicted using miRWalK (19871, FS; 13946, FSO; 8657, SDG) and their corresponding enriched pathways (15, FS; 2, FSO; 3, SDG). Fig. 1 visualizes these enriched pathways. Fig. 2 shows these enriched pathways, the miRWalK-identified genes involved (only those targeted by more than 3 miRNA for FS, for clarity), and corresponding miRNAs. Supplementary Fig. 1 shows significantly enriched pathways, all genes involved, and corresponding miRNA in the FS diet. Supplementary Tables 3 and 4 contain the predicted gene targets of these miRNA as determined by TargetScan: 1069 (FS), 2179 (FSO), and 935 (SDG) and their corresponding enriched pathways: 2 (FS), 12 (FSO), and 3 (SDG). No gene target predictions were found for miR-297 (FS diet) and miR-1966 (SDG diet). Supplementary Tables 5 and 6 contain the predicted gene targets as determined via miRDB: 6926 (FS), 4297 (FSO), and 2827 (SDG) and their corresponding enriched pathways: 9 (FS), 0 (FSO), and 1 (SDG), respectively.

## 2 Experimental Design, Materials and Methods

### 2.1 Animal study and experimental diets

The study design, diet composition and sample collection are available at [1] and [2]. Briefly, C57BL/6 mice were randomized into 4 diet groups to receive a basal diet (BD), or a diet modified to contain 10% FS, 3.67% FSO, OR 0.15% SDG (n=14/group). After 21 days, the fourth left mammary gland was excised from 6 mice per group (representing different cages and average body weight) and stored at -80˚C for RNA extraction and miRNA profiling [1]. Female mice were used in this study as previously established clinical benefits of flaxseed pertain to breast health and cancer in women.

### 2.2 RNA extraction and miRNA profiling

Details on the procedure utilized for RNA extraction and miRNA profiling are provided in the related manuscript [1]. For clarity, basic information is included here. Briefly, RNA was extracted using the miRVana^TM^ miRNA isolation Kit (Catalog No. AM1560, Ambion, Life Technologies, Waltham, MA, USA) and used to profile 578 miRNAs with the nCounter® Mouse v1.5 miRNA Expression Assay Kit (Catalog No. CSO-MMIR15-12, NanoString Technologies, Seattle, WA, USA) (miRBase built v15). As described in the related manuscript [1], raw data were processed using nSolver^TM^ Analysis Software v4.0 (NanoString Technologies). Samples with more than 25% of probes below the background, including one sample in the BD group and one sample in the FSO group, were excluded from the analysis, resulting in n=5 in these groups. Data were log2 transformed before being used for statistical analysis.

### 2.3 *In silico* gene target and pathway analysis

Predicted target genes of miRNAs were identified *in silico* with miRWalk Release 2.0 (binding p-value > 0.95) [3], TargetScanMouse release 8.0 (highly conserved miRNAs only) [4], and miRDB version 6 (Target Score > 50) [5]. For each diet, pathways significantly enriched by genes targeted by unique miRNAs were identified with pathDIP 4 (for mouse, ortholog pathways members, Bonferroni corrected p-value < 0.05) searching all 23 pathway sources available [6]. Using NAViGaTOR, pathways and their corresponding gene targets of miRNAs identified in miRWalk were visualized.

## Ethics Statements

All the animal procedures followed the ARRIVE guidelines and were approved by the animal ethics committee at the University of Toronto (Animal Use Protocol #: 20011734). All procedures were performed in accordance with the Regulations of the Animals for Research Act in Ontario and the Guidelines of the Canadian Council on Animal Care.

## CRediT Author Statement

**Diana Wu:** Writing – Original Draft, Writing – Review & Editing, visualization, investigation, formal analysis; **Amel Taibi:** Writing – Original Draft, Writing – Review & Editing, investigation, methodology, data curation; **Zhen Lin:** Methodology, investigation; **Lilian Thompson:** Supervision, Writing – Review & Editing, funding acquisition; **Elena Comelli:** Conceptualization, supervision, Writing – Review & Editing, funding acquisition, project administration.

## Declaration of Competing Interest

The authors declare that they have no known competing financial interests or personal relationships that could have appeared to influence the work reported in this paper.

The authors declare the following financial interests/personal relationships which may be considered as potential competing interests:

All funding agencies have been listed in the Acknowledgments section.

EMC has received research support from Lallemand Health Solutions and Ocean Spray and has received consultant fees, speaker, or travel support from Danone and Lallemand Health Solutions (All are outside of this work). The authors declare that they have no known competing financial interests or personal relationships which have or could be perceived to have influenced the work reported in this article.

## Data Availability

Data on mammary gland microRNA predicted gene targets and corresponding pathway analysis in female mice receiving flaxseed or its oil and secoisolariciresinol diglucoside components (Original data) (Harvard Dataverse). Mammary Gland microRNA profiling in female mice receiving flaxseed or its oil and secoisolariciresinol diglucoside components (Original data) (Gene Expression Omnibus (GEO)).

## Appendix Supplementary materials

Image, application 1

Image, application 2

Image, application 3

Image, application 4

Image, application 5

Image, application 6

Image, application 7
