# Supplementary material for: Data on mammary gland microRNAs expression, their predicted gene targets and corresponding pathway analysis in female mice receiving flaxseed or its oil and secoisolariciresinol diglucoside components
Source: Data Brief. 2022 May 29;42:108328. doi: 10.1016/j.dib.2022.108328 (PMC9167859; doi:10.1016/j.dib.2022.108328)
Supplement: Supplementary file 1 [file mmc1.docx]

*
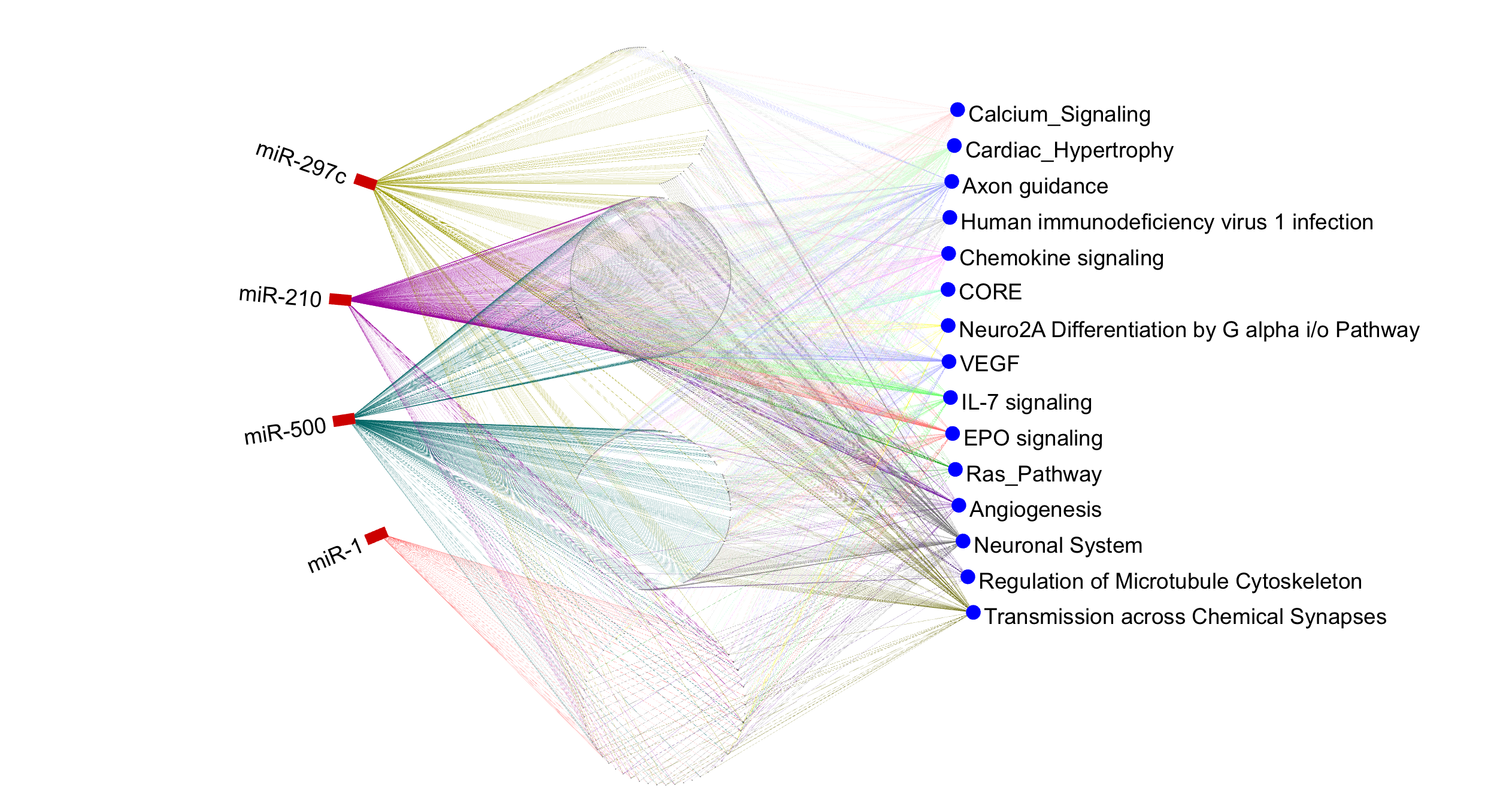
*

Supplementary Figure 1: Predicted miRNA-gene target-pathway (Bonferroni corrected p-value < 0.05) network. Gene targets were identified with mIRWalk for miRNAs unique to the FS diet. For interpretability, genes were not labelled. A full list of genes can be found in Supplementary Table 1. MiRNAs are denoted in red and pathways are denoted in blue.
